# Green-to-red photoconvertible fluorescent proteins: tracking cell and protein dynamics on standard wide-field mercury arc-based microscopes

**DOI:** 10.1186/1471-2121-11-15

**Published:** 2010-02-22

**Authors:** Susan M Baker, Robert W Buckheit, Matthias M Falk

**Affiliations:** 1 Department of Biological Sciences, Lehigh University, 111 Research Drive, Iacocca Hall, Bethlehem, PA 18015, USA; 2 Current address: National Center for Forensic Science, University of Central Florida, Orlando, FL, USA; 3 Current address: Johns Hopkins School of Medicine, Cellular and Molecular Medicine Graduate Training Program, Baltimore, MD 21231, USA

## Abstract

**Background:**

Green fluorescent protein (GFP) and other FP fusions have been extensively utilized to track protein dynamics in living cells. Recently, development of photoactivatable, photoswitchable and photoconvertible fluorescent proteins (PAFPs) has made it possible to investigate the fate of discrete subpopulations of tagged proteins. Initial limitations to their use (due to their tetrameric nature) were overcome when monomeric variants, such as Dendra, mEos, and mKikGR were cloned/engineered.

**Results:**

Here, we report that by closing the field diaphragm, selective, precise and irreversible green-to-red photoconversion (330-380 nm illumination) of discrete subcellular protein pools was achieved on a wide-field fluorescence microscope equipped with standard DAPI, Fluorescein, and Rhodamine filter sets and mercury arc illumination within 5-10 seconds. Use of a DAPI-filter cube with long-pass emission filter (LP420) allowed the observation and control of the photoconversion process in real time. Following photoconversion, living cells were imaged for up to 5 hours often without detectable phototoxicity or photobleaching.

**Conclusions:**

We demonstrate the practicability of this technique using Dendra2 and mEos2 as monomeric, photoconvertible PAFP representatives fused to proteins with low (histone H2B), medium (gap junction channel protein connexin 43), and high (α-tubulin; clathrin light chain) dynamic cellular mobility as examples. Comparable efficient, irreversible green-to-red photoconversion of selected portions of cell nuclei, gap junctions, microtubules and clathrin-coated vesicles was achieved. Tracking over time allowed elucidation of the dynamic live-cycle of these subcellular structures. The advantage of this technique is that it can be performed on a standard, relatively inexpensive wide-field fluorescence microscope with mercury arc illumination. Together with previously described laser scanning confocal microscope-based photoconversion methods, this technique promises to further increase the general usability of photoconvertible PAFPs to track the dynamic movement of cells and proteins over time.

## Background

Expression of GFP-fusion proteins in live cells revolutionized cell biology by allowing for visualization and tracking of proteins of interest in real time at high spatio-temporal resolution. A few years ago, encouraged by the successful use of GFP and of other fluorescent proteins, several laboratories began to develop photoactivatable and photoconvertible fluorescent proteins (PAFPs), which typically undergo a pronounced increase or shift in their spectral emission properties in response to UV-violet (350-420-nm) or, in case of Dendra2 also intense blue light (488 nm) illumination [1-4]. Monomeric *Anthozoa*-derived green-to-red photoconvertible fluorescent proteins (Dendra2, mEos2, mKikGR) showed particular promise for improved methods of tracking the dynamics of discrete protein pools within cells [5-8]. In the dark, these proteins mature to a green fluorescent state with a half-time of about 90 minutes at 37°C [7,9], while irradiation with UV-violet light (and also intense blue light in case of Dendra2) results in their irreversible transition into a very photostable, bright-red fluorescent state that can be tracked for hours and days without significant photobleaching on a fluorescence microscope [6,7,10-12].

Protein kinetics and rates of protein exchange are typically determined through the use of techniques such as fluorescence recovery after photobleaching (FRAP) or fluorescence loss in photobleaching (FLIP). However, these techniques are limited in that photobleached protein populations may not be tracked beyond the point of photobleaching. Obviously, photoconversion offers a distinct advantage over photobleaching, if continuous tracking of subpopulations of tagged proteins is desired.

**Figure 1:**
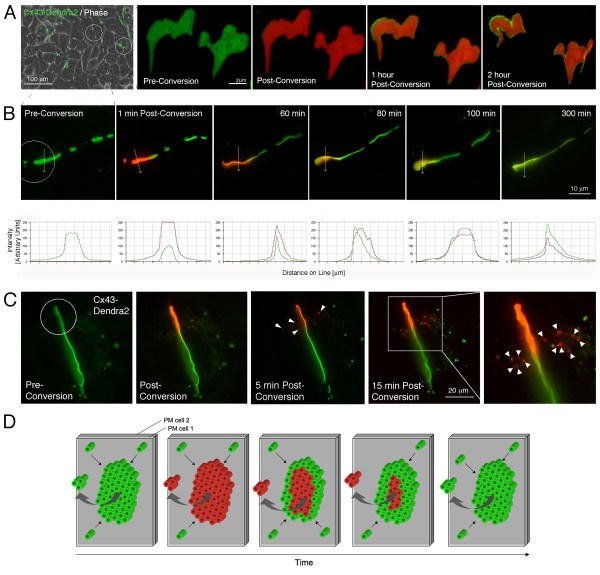
**Gap junction (GJ) dynamics revealed by photoconverting expressed Cx43-Dendra2**. **(A) **GJs consisting of many densely packed channels visible as green lines and puncta between HeLa cells expressing Cx43-Dendra2 (panel 1). GJs orient perpendicular providing a view onto their edge (circled in center, panel 1; also shown in Figures 1B, C), or horizontally providing a view onto their surface (circled on right; remaining panels, Figure 1A). GJs are dynamic structures. Their channels are replaced within several hours, as demonstrated by photoconverting Dendra2-tagged Cx43. A region containing two horizontally oriented GJs was photoconverted (entire field shown), and green and red channels were recorded over time. Within 1-hour post conversion a widening, homogenous green line of channels appeared along the GJs (panels 2-5). **(B) **Following photoconverted GJs for longer periods resulted in a steady loss of red fluorescence from the photoconverted area, and a simultaneous recovery of green fluorescence (circled in panel 1), suggesting that older channels are continuously removed from central GJ areas, while newly synthesized channels are simultaneously added to their periphery. Fluorescence intensity profiles for red and green channels measured along lines traversing the photoconverted GJs are shown. **(C) **Photoconversion allows estimation of GJ channel turnover. A portion of a perpendicular oriented Cx43-Dendra2 GJ was photoconverted (circled). Over time, red fluorescent puncta appeared adjacent to the converted GJ area (arrow-heads, panels 3, 4). Puncta were not detected immediately post-conversion (panel 2), suggesting that they were released from the photoconverted GJ area. Puncta correspond to degradative endocytic vesicles that are generated by the release of small GJ channel packets from GJs [15]. **(D) **Schematic representation of GJ turnover as shown experimentally in (A-C).

Over the past decade, we have used green fluorescent protein (GFP) fusions and other color variants to track the dynamics of gap junction (GJ) channels in living cells, including by FRAP and FLIP techniques [13-19]. GJ channels assemble from hexamers of the four-pass transmembrane proteins called connexins (Cxs) that cluster together into so-called "plaques" in the lateral plasma membranes of cells to provide direct cell-to-cell communication and physical cell-cell coupling (Figure 1A, panel 1). GJ plaques can be aligned in two principal orientations: perpendicular to the image plane, providing a view onto their edge (GJ plaques circled in the center of Figure 1A and shown in Figures 1B, C); or horizontally, if cells grow partially on top of each other, providing a view onto their surface (en face) (GJ plaques circled on the right of Figure 1A and shown in the remaining panels of Figure 1A). A typical GJ plaque can consist of hundreds to thousands of densely packed individual channels. GJs are surprisingly dynamic membrane structures. Their structural connexin proteins have been found to turn over with a half-life of only 1 to 5 hours [15,20-22]. Tagging of Cx-proteins with tetrameric photoconvertible fluorescent proteins, such as DsRed was found not to be feasible, probably due to the disruption of appropriate Cx-oligomerization by the tetrameric FP tag [13,23]. However, monomeric fluorescent protein tags such as GFP, mCherry, mEos2, or Dendra2 are tolerated by many different proteins without detectable interference with their function, including Cxs [3,4,6,7,10,14,15,24-26]. We report an efficient technique that makes photoconversion and tracking of discrete protein pools feasible on simpler, less expensive mercury arc-based fluorescence microscopes equipped with standard fluorescence filter sets that complements previously published more sophisticated laser scanning confocal microscope-based techniques [3,4,6,7,10,12,26,27]. We demonstrate the feasibility of this technique using Dendra2-tagged histone H2B (Dendra2-H2B), connexin43 (Cx43-Dendra2), α-tubulin (Dendra2-α-tubulin), and clathrin light chain (mEos2-clathrin light chain) as examples of proteins exhibiting a wide array of dynamic properties. A detailed step-by-step photoconversion protocol is provided.

## Results and Discussion

**Figure 2:**
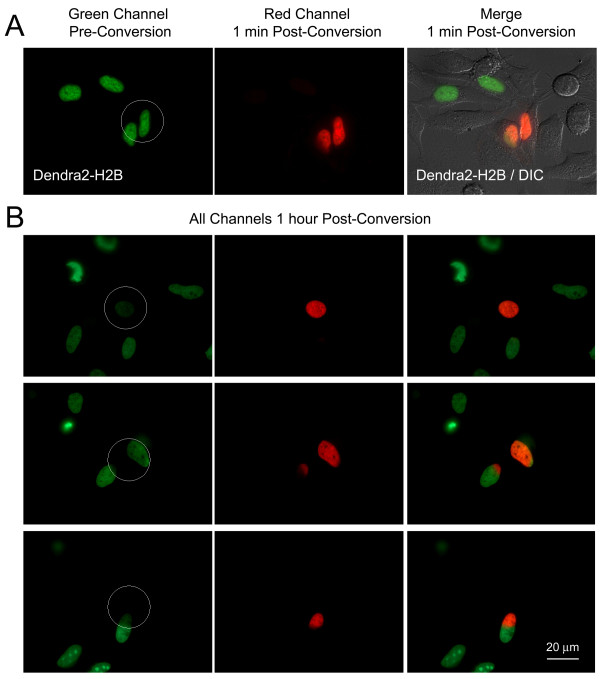
**Histone 'dynamics' revealed by photoconverting expressed Dendra2-H2B**. Entire cell nuclei (in A, and top row in B), or portions of nuclei (center and bottom rows in B) of Dendra2-H2B expressing HeLa cells were photoconverted within 5-10 sec (circled areas), and green and red channels were imaged immediately after photoconversion (in A), and after 1-hour (in B). One-hour post conversion, the areas of photoconverted histone H2B protein and the edges between photoconverted and unconverted Dendra2-H2B domains were still well defined, revealing H2B's stable association with DNA in interphase chromatin. DIC and fluorescence images were merged in (A) to reveal the location of cell nuclei, and transiently transfected Dendra2-H2B expressing cells. Note that more or less efficient photoconversion of cell nuclei was achieved (compare remaining green fluorescence in the circled areas in B, rows 1 and 3, compared with row 2).

**Figure 3:**
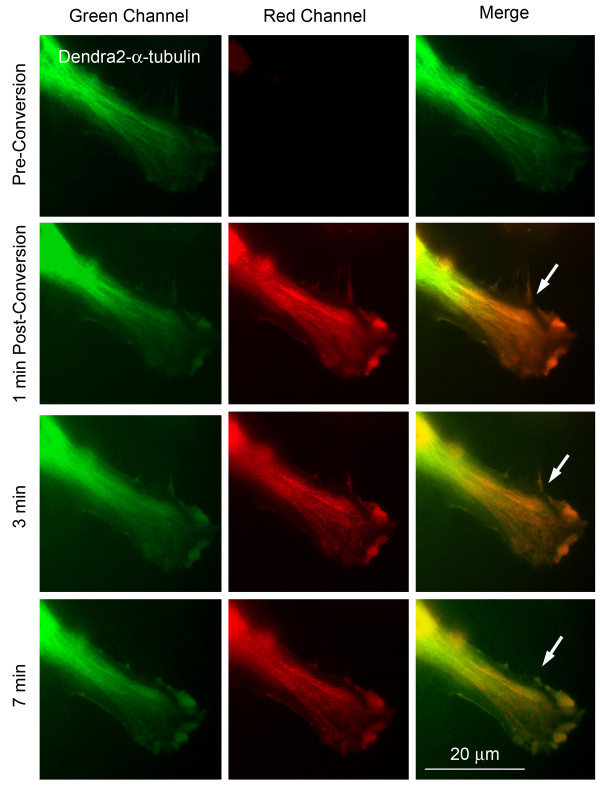
**Microtubule dynamics revealed by photoconverting expressed Dendra2-α-tubulin**. A distal portion of a Dendra2-α-tubulin expressing HeLa cell was photoconverted within 5 sec (entire field shown in the panels). Note, that photoconversion was only incomplete, as indicated by the relatively strong remaining green fluorescence (visible in the left panel of row 2). Within 3-7 minutes, red photoconverted and remaining green α-tubulin pools intermixed (arrows), consistent with the known dynamic continuous polimerization and depolimerization of microtubules, and the diffusional mobility of unassembled α/β-tubulin subunit-dimers in the cytoplasm.

**Figure 4:**
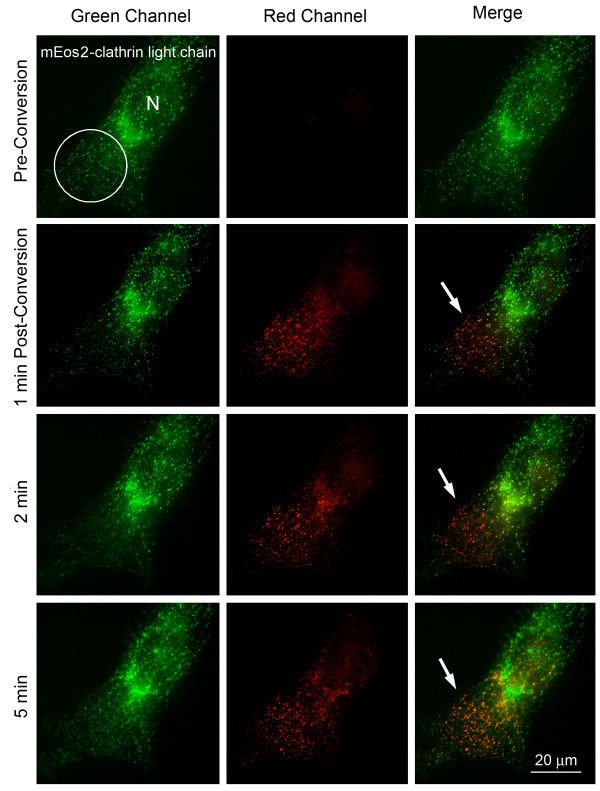
**Clathrin-coated vesicle dynamics revealed by photoconverting expressed mEos2-clathrin light chain**. Numerous clathrin-coated vesicles are visible in the cytoplasm of an mEos2-clathrin light chain expressing HeLa cell (panel 1, row 1). A distal portion of this vesicle pool was photoconverted within about 10 sec (circled). Within 2-5 minutes after photoconversion, photoconverted and remaining, unconverted vesicles moved laterally and intermixed (red, green and yellow vesicles, the resulting color when red and green fluorescence colocalize, arrows), consistent with the known dynamic mobility and structural composition of these vesicles. N = Cell nucleus.

To demonstrate the feasibility of photoconverting and tracking discrete subcellular PAFP-tagged protein pools on a wide-field fluorescence microscope, we generated and used cDNA constructs in which Dendra2 or mEos2, two available comparable monomeric PAFPs, were fused to the C-terminus of the GJ protein Cx43, or to the N-termini of histone H2B, α-tubulin and clathrin light chain [4,15,26]. In transiently transfected HeLa cells, we observed expression, assembly, trafficking and localization of all four fusion-protein constructs similar to untagged, or GFP-tagged fusion constructs as reported previously [4,14,25,26] (Figures 1, 2, 3 and 4). Using 450-490 nm mercury arc lamp illumination (standard FITC or GFP-filter sets), significantly attenuated by neutral density filters (ND8/ND4), we were able to visualize and image green Dendra2- and mEos2-fluorescence without unintentionally photoconverting these PAFPs from green to red fluorescence (Figures 1, 2, 3 and 4).

Dendra2/mEos2 fusion-protein pools underwent precise and selective photoconversion by moving the target areas to the center of the visible field, closing the field diaphragm to pinhole size (exposing an area as small as 20 μm diameter using a 100× objective), and exposing the areas to full, 330-380 nm illumination (standard DAPI filter set) for 5-10 seconds. Use of a DAPI-filter cube with 420 nm long-pass emission filter (LP420) allowed to observe and manipulate the photoconversion process in real time. Following photoconversion, filters were switched to the red fluorescence filter cube (standard Rhodamine filter set; excitation: 530-560 nm; emission: 590-650 nm), the field diaphragm was re-opened completely, and the red photoconverted Dendra2 signal was detected and imaged using the red filter cube. Depending on exposure time, up to 90% of the green Dendra2/mEos2 fluorescence in the target area was photoconverted into red fluorescence within a few seconds (Figures 1, 2, 3 and 4). Green fluorescence outside the target area was not converted (Figures 1, 2, 3 and 4). Subsequent to photoconversion, cells were imaged repeatedly in both green (attenuated by neutral density filters to prevent further unintentional photoconversion) and red channels at indicated time intervals for up to 5 hours (Figures 1, 2, 3 and 4).

Over a 5-hour period, in live cells expressing Cx43-Dendra2, we observed a significant loss of red fluorescence from the target area and recovery of the green fluorescence signal (Figure 1B), indicating the continuous removal of older, red photoconverted Cx43-Dendra2-based GJ channels and delivery of newly synthesized, green Cx43-Dendra2 channels to GJ plaques (Figure 1D), as observed previously for GJs using other fluorescence based techniques [15,17,28]. To confirm that photoconversion was irreversible and stable, and we were not observing theoretical auto-recovery of green Dendra2-fluorescence, we fixed Cx43-Dendra2 expressing cells in formaldehyde following photoconversion. No recovery of green fluorescence was observed hours, or even days post conversion (not shown).

To track accrual of new channels to GJ plaques, a 20 μm diameter region (using a 100× objective and maximally closed field-diaphragm) encircling two Cx43-Dendra2 GJ plaques was photoconverted from green to red fluorescence (Figure 1A, panel 2, 3) and imaged every 30 minutes for 2 hours. After 1 hour, a distinct rim of newly accrued (green) channels had appeared along the edges of the photoconverted (red) GJ plaques (Figure 1A, panel 4) that became wider and more intense over time (Figure 1A, right panel), indicating that new channels are indeed accrued along the outer edges of GJ plaques, while older channels are simultaneously removed from plaque centers as schematically shown in Figure 1D and in references [15,17]. These findings are consistent with what has previously been reported for channel accrual to GJ plaques using fluorescence recovery after photobleaching (FRAP) and successive FlAsH and ReAsH labeling techniques [17,28].

To investigate whether this technique would allow estimation of protein turnover kinetics, a portion of a perpendicular oriented Cx43-Dendra2 GJ plaque was photoconverted from green to red fluorescence as described above (Figure 1C, left panel, circled area) and imaged every 5 minutes. Over time, we detected red fluorescent puncta in the cytoplasm adjacent to photoconverted plaques that accumulated over the 15 min time period (Figure 1C, panels 3, 4, marked with arrow-heads). The red fluorescent puncta we recognized as degradative endocytic vesicles that were released from the photoconverted GJ plaque area and later were degraded by lysosomal pathways [15] (not shown). These red fluorescent vesicles were not detected immediately post-photoconversion (Figure 1C, panel 2), suggesting that they were not already present in the cytoplasm at the time of photonversion. Calculating surface areas of released vesicles revealed a half-life of ~2.5 hours (approximately 10 μm^2 ^of a 50 μm^2 ^photoconverted GJ plaque area was internalized in one hour) [15] that falls within the estimated half-life of 1-5 hours reported previously for GJs [20-22]. While certainly possible on wide-field microscopes, quantitative photoconversion experiments depending on the target, may however better be performed on confocal microscope systems, that allow to precisely restrict the photoconversion laser beam to the target area without potentially photoconverting unintentionally other structures, that are also located within the circular wide-field microscope photoconversion area [15].

Histones H2A, H2B, H3, and H4 are the core protein components of nucleosomes and are known to be bound stably to interphase chromatin; and this was confirmed by the appearance of the photoconverted red Dendra2-histone-H2B fluorescence that stayed locally unchanged over time in the cell nuclei of living, Dendra2-histone-H2B-expressing HeLa cells imaged before, immediately after, and 1 hour after photoconversion (Figures 2A, B).

In contrast, clathrin and tubulin are known to be highly dynamic cellular proteins and this was confirmed in our photoconversion experiments. Microtubules are assembled from α/β-tubulin dimers and both populations, assembled microtubules as well as unassembled cytoplasmically located tubulin dimers (diffuse green fluorescence), are visible in living Dendra2-α-tubulin expressing Hela cells (Figure 3, top row). In addition, microtubules are known to constantly grow and shrink, resulting in a dynamic exchange of subunits in an assembled microtubule. Within a few minutes after a distal portion of a Dendra2-α-tubulin expressing HeLa cell was partially photoconverted (Figure 3, second row, arrows), red photoconverted and remaining green α-tubulin pools were observed to intermix, consistent with the known dynamic growth behavior of microtubules, and the mobility of unassembled subunits in the cytoplasm. Tubulin dynamics resulted in a diffusion of red fluorescence away from the distal, photoconverted area and an increasing re-occurrence of unconverted green α-tubulin in the distal cell area within several minutes after photoconversion (Figure 3, rows 3, 4).

Clathrin triskelions consisting of three light and three heavy chains assemble into flat, and curved lattices on the plasma membrane to build the coats of endocytic vesicles. Numerous such vesicles are visible in living HeLa cells expressing mEos2-clathrin light chain (Figure 4, top row). Within 5 minutes after a distal portion of this vesicle pool in a cell was successfully photoconverted (circled in Figure 4, panel 1; rows 2-4, arrows), red photoconverted and remaining green vesicles were observed to move laterally and to intermix with each other, consistent with the dynamic nature of these vesicles that are trafficked throughout the cell along microtubules (Figure 4, bottom row).

While several studies have described photoconversion of PAFPs using laser scanning confocal microscopy [6,7,9-11,15,26], we describe here PAFP-photoconversion of discrete protein pools using a simpler, less expensive mercury arc-based microscope system. As such, there are restrictions presented by photoconverting PAFPs on a standard wide-field fluorescence microscope as opposed to a laser scanning confocal microscope system. First, the smallest region that can be photoconverted is limited in size and shape to a circular area of about 20 μm (using a 100× objective; about 35 μm using a 60× objective) that is defined by the minimal remaining opening of the field diaphragm. However, aftermarket 10 μm diameter pinhole- and slit-sliders are available to partially overcome this limitation (e.g. slit/pinhole slider for Olympus inverted microscopes; Melles Griot Inc., Albuquerque, NM, USA).

Since photon emission of mercury arc bulbs is less intense compared to lasers, photoconversion on mercury-arc lamp based microscope systems takes longer (several seconds at UV-violet excitation), and photoconversion might remain incomplete (Figures 1, 2, 3 and 4). Prolonged exposure to short-wavelength light can be detrimental to living cells that may react by inducing apoptosis in response to toxic short-wavelength illumination (e.g. DAPI 330-380 nm band-pass excitation filter). Thus, the addition of HEPES-buffer and Oxyrase (Oxyrase Inc., Mansfield, Ohio, USA) to the cell-culture medium to degrade toxic oxygen radicals generated during short-wavelength excitation is recommended. Also, Dendra2 and mEos2's major photoconversion wavelength is 405 nm [7,26] and optical filters allowing specific excitation at this red-shifted, less harmful wavelength are commercially available (Chroma, Omega, Semrock). On the other hand, due to the less intense mercury arc lamp illumination, photobleaching instead of photoconversion that will occur on confocal microscope systems if laser power is set too high is not problematic. Furthermore, Dendra2 (but not mEos2) has a second less efficient photoconversion peak at 490 nm [7,26] that renders green Dendra2 sensitive to intense blue light; and allows Dendra2 (but not mEos2) to be photoconverted on standard Argon-Helium/Neon laser-equipped confocal microscopes. Thus, when imaging green Dendra2, illumination attenuated by a neutral density filter (or very low 488 nm laser power) is required to avoid inadvertent photoconversion. However, if phototoxicity due to UV-violet illumination should be a problem, less efficient, but marginally toxic un-attenuated FITC-filter illumination (450-490 nm excitation band pass filter) can be used to photoconvert Dendra2, instead of using the DAPI filter set.

## Conclusions

We demonstrate here efficient and irreversible photoconversion of discrete subcellular protein pools of cells grown in culture on wide-field fluorescence microscopes, equipped with standard filter cubes and mercury arc-lamp illumination, using several Dendra2- and mEos2-tagged proteins with a wide variety of dynamic properties as examples. Dendra2 and mEos2 behaved comparable in respect of time required for photoconversion (5-10 sec), and conversion efficiency (30-90%). Photobleaching during photoconversion, and afterwards during repeated imaging, was not observed as being problematic. The advantage of this method is its greater simplicity requiring easier to use, less expensive microscope systems that makes this technique especially appealing to less well equipped institutions, for instructional and teaching purposes, and to applications where defining a region of interest of specific size or shape (e.g. whole cell applications) is not required. Disadvantages to laser scanning confocal microscope-based photoconversion techniques include the limited control of the region to be photoconverted (circular, ≥ 20 μm in diameter), and longer, potentially more toxic UV-violet photoconversion times. Together with previously described laser scanning confocal microscope-based photoconversion methods, this technique promises to further increase the general usability of photoconvertible PAFPs to track the dynamic movement of cells and proteins over time. This technique may also be applicable to locally un-cage fluorescent probes, activate paGFP, or to study the effects of locally induced photo-damage.

## Methods

### Plasmid construction for expression in mammalian cells

For expression in eukaryotic cells, a *Bam*HI-*Eco*RI fragment of cDNA encoding Cx43 described in ref [14] was inserted into the pDendra2-N1 plasmid. The cDNA endcoding Cx43 was obtained from a *BamH1-EcoRI *digest of a Cx43-pEGFP-N1 plasmid. The pDendra2-N1 vector, the Dendra2-H2B, the Dendra2-human α-tubulin, and the mEos2-clathrin light chain constructs were generous gifts of Michael W. Davidson (Florida State University, Tallahassee, FL).

### Cell culture and transfection

HeLa cells (CCL 2, American Type Culture Collection, Manassas, VA) were cultured under standard culture conditions as described previously [14]. For all experiments, cells were grown on 35 mm diameter glass bottomed culture dishes coated with 20 μg/mL collagen (Mat-Tek Corp., Ashland, MA). Cells were transfected using Superfect transfection reagent (Qiagen, Valencia, CA) according to manufacturer's recommendations 24 hours prior to photoconversion experiments. All photoconversion experiments were carried out at 37°C in standard culture medium supplemented with HEPES (15 μg/mL) (Sigma) and oxyrase (40 μL/mL) (Oxyrase Inc.) in an enclosed environmental control chamber enclosing the microscope.

### Fluorescence microscopy and photoconversion

Time-lapse microscopy was performed on a Nikon Eclipse TE 2000E inverted fluorescence microscope equipped as previously described [19]. Photoconversion was performed by reducing the field diaphragm to pinhole size, at 100× magnification with near-UV irradiation (330-380 nm) for 5-10 seconds. Photoconversion was monitored in real time using a DAPI filter cube with long-path emission filter (LP420). Similarly efficient photoconversion (30-90%) was observed with both Dendra2 and mEos2 PAFPs. Images were captured and analyzed using MetaVue software version 6.1r5 (Molecular Devices, Sunnyvale, CA) and processed using Adobe Photoshop (Adobe Systems, Mountain View, CA). Unintentional photoconversion by room-light was not observed to be problematic, and cells -previous to photoconversion- where handled under standard cell culture conditions (e.g. dishes were not wrapped in aluminum foil).

#### Step-by-step procedure to photoconvert Dendra2- and mEos2-tagged protein pools on wide-field fluorescence microscopes

##### Steps to be done before photoconversion

1) Darken the room completely and dark-adapt your eyes.

2) Rotate filter cubes to blue light excitation position (FITC filter cube).

3) Push neutral density filters into light path.

4) Close excitation shutter on the microscope.

5) Rotate 40×, 60×, or 100× oil immersion objective into position and add immersion oil.

6) Place dish with Dendra2/mEos2-expressing cells on the stage.

7) Focus cells using white-light Phase Contrast or DIC illumination.

8) Open excitation shutter and search for expressing cells using strongly attenuated light only!

9) Acquire an image with attenuated blue excitation light (**pre-conversion green image**).

10) Rotate filter cubes to green light excitation position (TRITC filter cube), pull out neutral density filters and acquire another image (**pre-conversion red image**). There should be no, or only minimal red Dendra2-emission signal visible. If there is a strong red signal visible, accidental photoconversion of the field of few has already occurred, and a new region of interest needs to be selected.

11) Push neutral-density filters back into the light path.

##### For photoconversion the following steps need to be performed

12) Rotate filter cubes to the DAPI cube position (UV excitation), pull out neutral density filters and watch the photoconversion process through the oculars (developing red, photoconverted Dendra2/mEos2-emission is visible if DAPI cube is equipped with 420 nm long path emission filter). This takes about 5-10 seconds to achieve 30-90% efficient photoconversion.

13) Push neutral density filters back into the light pass to end the photoconversion process.

14) Acquire post-conversion images with blue (FITC cube) and red (TRITC cube) light illumination under the same conditions used for pre-conversion images. A strong red Dendra2-emission signal should now be visible.

15) To restrict photoconversion to the center of the field of view, place region of interest in the center of the viewable field. Close the field diaphragm to the desired position before photoconversion. Photoconvert region of interest. Re-open field diaphragm after photoconversion has been terminated.

16) Acquire green and red post-conversion images at desired time intervals under the conditions described above.

17) Merge green and red post-conversion images if desired.

## Abbreviations

Cx: connexin; FLIP: fluorescence loss in photobleaching; FRAP: fluorescence recovery after photobleaching; GFP: green fluorescent protein; GJ: gap junction; LP filters: long pass filters; ND filters; neutral density filters; PAFP: photoactivatable fluorescent proteins; UV: ultra-violet.

## Authors' contributions

MMF conceived and designed the study and the experiments; SMB, RWB, and MMF performed the experiments and analyzed the data; SMB and MMF drafted and wrote the manuscript and assembled and generated the figures. All authors read and approved the final manuscript.
